# A novel isoprimeverose‐producing enzyme from *Phaeoacremonium minimum* is active with low concentrations of xyloglucan oligosaccharides

**DOI:** 10.1002/2211-5463.12549

**Published:** 2018-12-11

**Authors:** Tomohiko Matsuzawa, Akihiko Kameyama, Katsuro Yaoi

**Affiliations:** ^1^ Bioproduction Research Institute National Institute of Advanced Industrial Science and Technology (AIST) Tsukuba Ibaraki Japan; ^2^ Biotechnology Research Institute for Drug Discovery National Institute of Advanced Industrial Science and Technology (AIST) Tsukuba Ibaraki Japan

**Keywords:** GH3, glycoside hydrolase, isoprimeverose, oligosaccharide, xyloglucan

## Abstract

Xyloglucan is one of the major polysaccharides found in the plant cell wall and seeds. Owing to its complex branched structure, several different hydrolases are required to degrade it. Isoprimeverose‐producing enzymes (IPase) are unique among the glycoside hydrolase 3 family in that they recognize and release a disaccharide from the nonreducing end of xyloglucan oligosaccharides. Only two IPases have been previously isolated and characterized. A novel IPase from *Phaeoacremonium minimum* (PmIPase) was expressed and characterized. The xylopyranosyl residue at the nonreducing end of xyloglucan oligosaccharides was essential for hydrolytic activity, and PmIPase was unable to hydrolyze cellobiose into d‐glucose. PmIPase had a *K*_m_ for xyloglucan oligosaccharide substrate that was much lower than that of the reported IPase isolated from *Aspergillus oryzae*. This indicates that PmIPase was able to produce isoprimeverose efficiently from low concentrations of xyloglucan oligosaccharides. PmIPase also exhibited transglycosylation activity and was able to transfer isoprimeverose units to its substrates.

**Abbreviations**
CBM: cellulose‐binding module
GH: glycoside hydrolase family
G: unbranched β‐d‐glucopyranosyl residue
IPase: isoprimeverose‐producing enzyme
L: β‐d‐galactopyranosyl‐(1→2)‐α‐d‐xylopyranosyl‐(1→6)‐β‐d‐glucopyranosyl segment
XXXGol: reduced XXXG substrate
X: α‐d‐xylopyranosyl‐(1→6)‐β‐d‐glucopyranosyl segment

Xyloglucan plays important roles in plant growth and development and is one of the major polysaccharides found in the plant cell wall and seeds 1. Xyloglucan is composed of a β‐1,4‐linked glucan backbone with glucopyranosyl moieties modified with α‐1,6 linked xylopyranosyl residues. Some of these xylopyranosyl residues are modified with additional saccharides, such as d‐galactose and l‐fucose 2. The structure of xyloglucan can be given as a series of abbreviations, where G represents an unbranched β‐d‐glucopyranosyl residue, X is an α‐d‐xylopyranosyl‐(1→6)‐β‐d‐glucopyranosyl segment, and L is a β‐d‐galactopyranosyl‐(1→2)‐α‐d‐xylopyranosyl‐(1→6)‐β‐d‐glucopyranosyl segment (Fig. 1) 3. Because of its complex branched structure, xyloglucan is degraded by several hydrolases. For example, xyloglucanases are xyloglucan‐specific endo‐β‐glucanases 4, 5, 6, 7, 8, and oligoxyloglucan reducing‐end‐specific cellobiohydrolases release two glucosyl main‐chain residues 9. α‐Xylosidases 10, 11, 12, isoprimeverose‐producing enzymes (IPase) 13, 14, 15, and β‐galactosidases 16 are also involved in xyloglucan degradation. Previously, it was reported that some commercial enzymes, such as Driselase (Sigma‐Aldrich, St. Louis, MO, USA) 17, and culture supernatant of *Aspergillus oryzae* 18 had isoprimeverose‐producing enzymatic activities. Only two IPases have been isolated, one from a bacterium (*Oerskovia* sp. Y1) 13 and the other from a eukaryote (*A. oryzae*) 15. The *A. oryzae* IPase, called IpeA, releases isoprimeverose (α‐d‐xylopyranosyl‐(1→6)‐d‐glucopyranose) units from xyloglucan oligosaccharides. The xylopyranose residue at the nonreducing end of xyloglucan oligosaccharide is essential for IpeA activity, and galactosylation of xylopyranose reduces IpeA activity 15. IPases exhibit both hydrolytic and transglycosylation activities and are able to produce di‐ and oligosaccharides 13, 15. Based on amino acid sequences, IPases belong to glycoside hydrolase family 3 (GH3; carbohydrate‐active enzymes database, http://www.cazy.org/). Most GH3 enzymes recognize and release monosaccharides from the nonreducing end of substrates. However, IPases recognize and release disaccharides. Despite the unique enzymatic properties of IPases, only two such enzymes have been isolated and characterized. The characterization of novel IPases may lead to a better understanding of xyloglucan degradation and GH3 enzymes. In addition, IPases can potentially produce unique oligosaccharides from lignocellulosic biomass. Xyloglucan oligosaccharides have been reported to have biological activities for lipid metabolism, ultraviolet‐induced immune suppression, and other processes 19, 20. Production of xyloglucan oligosaccharides, including isoprimeverose, is expected to contribute to the discovery of novel applications of xyloglucan oligosaccharides and effective utilization of lignocellulosic biomass. In this study, we expressed and characterized a novel IPase from *Phaeoacremonium minimum* (also called *Togninia minima*). *Phaeoacremonium minimum* belongs to Sordariomycetes and is frequently isolated from diseased woody plants 21. *Phaeoacremonium minimum* isoprimeverose‐producing enzyme (PmIPase) hydrolyzes and releases isoprimeverose from the nonreducing end of xyloglucan oligosaccharides. The *K*_m_ of PmIPase for a reduced XXXG substrate (XXXGol) was approximately one‐thirteenth that of *A. oryzae* IpeA, and PmIPase was able to produce isoprimeverose at low concentrations of substrate.

**Figure 1:**
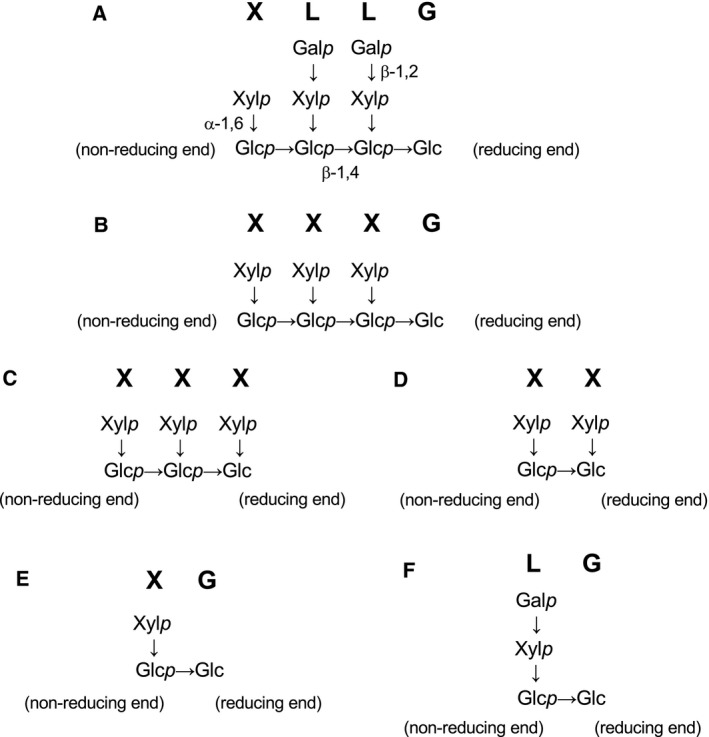
Structures and abbreviations of the xyloglucan oligosaccharides used in this study. (A) XLLG, (B) XXXG, (C) XXX, (D) XX, (E) XG, and (F) LG. Glc*p*, d‐glucopyranosyl residue; Xyl*p*, d‐xylopyranosyl residue; Gal*p*, d‐galactopyranosyl residue. Abbreviations are shown in boldface. In this paper, ‘nonreducing end’ or ‘reducing end’ indicates the nonreducing end or reducing end of the main chain of a xyloglucan oligosaccharide, respectively.

## Materials and methods

### Materials

Xyloglucan oligosaccharides were prepared as described previously 15. Figure 1 shows the structures of the xyloglucan oligosaccharides used in this study. Tamarind seed xyloglucan was purchased from Megazyme (Wicklow, Ireland), cellobiose from Sigma‐Aldrich, and xylobiose from Wako Pure Chemical Industries (Osaka, Japan).

### Expression and purification

The gene encoding PmIPase was synthesized by GENEWIZ (South Plainfield, NJ, USA) with codon optimization for *Pichia pastoris*, and the synthesized DNA sequence was deposited in DDBJ/EMBL/GenBank under the accession number LC333973. The synthesized PmIPase gene was digested with *Eco*RI and *Not*I and ligated to a pGAPZα A vector (Invitrogen, Carlsbad, CA, USA) digested with *Eco*RI and *Not*I. pGAPZα A‐PmIPase‐Myc‐ and His_6_‐tag vector was treated with *Avr*II and introduced into *P. pastoris* strain X‐33. *Pichia pastoris* X‐33 cells harboring PmIPase‐Myc‐ and His_6_‐tag were cultured in YPD medium (2% peptone, 1% yeast extract, and 2% d‐glucose) containing 50 mm potassium phosphate buffer (pH: 6.0) at 30 °C, 100 r.p.m. for 3 days. After cultivation, cells were removed by centrifugation (5000 ***g***, 15 min) and filtration (0.22 μm). Recombinant PmIPase was purified using a Ni^2+^‐affinity column (HisTrap FF; GE Healthcare, Buckinghamshire, UK). The culture supernatant was run through a HisTrap FF column and washed several times with buffer containing 20 mm sodium phosphate (pH: 7.4), 300 mm NaCl, and 10 mm imidazole. The recombinant PmIPase was eluted with buffer containing 20 mm sodium phosphate (pH: 7.4), 300 mm NaCl, and 500 mm imidazole. The purified enzyme was concentrated using a Vivaspin 20–10k cut (GE Healthcare) ultrafiltration system, and the protein concentration was measured by UV (280 nm) absorbance using a NanoDrop spectrophotometer (NanoDrop Technologies, Rockland, DE, USA) and revised using ProtParam (http://web.expasy.org/portparam/). Briefly, the protein concentration measured using the NanoDrop spectrophotometer was divided by the extinction coefficient from ProtParam. Recombinant *A. oryzae* IpeA was expressed and purified as described previously 15.

### Endoglycosidase treatment

Purified recombinant PmIPase was treated with endoglycosidase H (endo H; New England Biolabs, Ipswich, MA, USA) following a slightly modified version of the method described previously 15. Four micrograms of purified recombinant PmIPase was denatured at 98 °C for 10 min in the presence of 0.5% SDS and 40 mm DTT. Then, 50 mm sodium phosphate buffer (at final concentration, pH: 6.0) and 1000 units of endo H were added to the reaction mixture. The reaction mixture was incubated at 37 °C for 60 min.

### Optimal temperature and pH

The optimal pH range of PmIPase was determined as described below. Twenty microliters of reaction mixture containing 0.3 μg of purified recombinant PmIPase, McIlvaine's buffer 22, and 7.5 mm XXXGol 13, 23 was incubated at 60 °C for 5 min. The resulting reducing sugars were measured using a dinitrosalicylic acid (DNS) reagent method 24.

The optimal temperature of PmIPase activity was determined as described below. Twenty microliters of reaction mixture containing McIlvaine's buffer (pH: 3.5), 0.3 μg of purified recombinant PmIPase, and 7.5 mm XXXGol was incubated at temperatures between 40 and 65 °C for 5 min. The released sugars were measured using the DNS method described above. The thermostability of PmIPase was determined by incubating the enzyme (0.06 mg·mL^−1^) at 40–65 °C in 50 mm sodium acetate buffer (pH: 4.0) for 5 min. After the heat treatment, 12.5 mm XXXGol was added and the reaction mixture was incubated at 60 °C for 5 min. The residual activity of heat‐treated PmIPase was measured using the DNS methods, as described above.

### Substrate specificity

Aliquots of the reaction mixture (50 μL) containing oligosaccharides (XG, LG, XX, XXX, XXXG, XLLG, XXXGol, cellobiose, and xylobiose), 50 mm sodium acetate buffer (pH: 4.0), and 0.1 μg of purified recombinant PmIPase were incubated at 60 °C for 5 min. To stop the reaction, mixtures were incubated at 98 °C for 10 min. The released isoprimeverose, d‐glucose, and d‐xylose were measured by HPLC system driven by a pump (PC‐2080; JASCO, Tokyo, Japan) and equipped with a refractive index detector (RI‐2031; JASCO) using an Aminex HPX‐87H 300 × 7.8‐mm column (Bio‐Rad, Hercules, CA, USA) as described previously 15.

### Kinetic analysis of recombinant PmIPase

The kinetic parameters (*K*_m_, *k*_cat_, and *k*_cat_/*K*_m_) of recombinant PmIPase for XXXGol were determined as described below. Twenty microliters of reaction mixture containing 50 mm sodium phosphate buffer (pH: 4.5), purified recombinant enzyme (1 ng of PmIPase or 0.5 ng of IpeA), and XXXGol (0.0125–0.8 mm for PmIPase; 0.0625–4 mm for IpeA) was incubated at 60 °C for 5 min. To stop the reaction, the mixture was incubated at 98 °C for 10 min. The resulting reducing sugars were measured using a bicinchoninate assay 25. A standard curve was constructed using isoprimeverose. Kinetic constants were calculated using a nonlinear regression of the Michaelis–Menten equation in graphpad prism version 5.0 (GraphPad Software, La Jolla, CA, USA).

### Transglycosylation activity

The transglycosylation activity of PmIPase was examined as described below. Fifty microliters of a reaction mixture containing 8 mm XXXG, 50 mm sodium acetate buffer (pH: 4.5), and 2 μg of purified recombinant PmIPase was incubated at 60 °C for 5 min. To stop the reaction, the mixture was incubated at 98 °C for 10 min. The reaction products were analyzed with HPLC system driven by a pump (LC‐20AD; Shimadzu, Kyoto, Japan) and equipped with a refractive index detector (RID‐20A; Shimadzu) using a TSKgel Amide‐80 5 μm column (4.6 mm I.D. ×25 cm; Tosoh, Tokyo, Japan), with 60% acetonitrile as the column eluent at a flow rate of 0.8 mL·min^−1^ at 40 °C.

### Mass spectrometry

The mass spectra were acquired using a MALDI‐TOF mass spectrometer (Ultraflex TOF/TOF; Bruker Daltonik, Bremen, Germany). Ions were generated using a pulsed 337‐nm nitrogen laser and were accelerated to 23.5 kV. All spectra were obtained in the linear mode with a delayed extraction of 60 ns. For sample preparation, 0.5 μL of a matrix solution prepared by dissolving sodium 2,5‐dihydroxybenzoate (1 mg·mL^−1^) and 2,5‐dihydroxybenzoic acid (19 mg·mL^−1^) in 30% ethanol was spotted onto a target plate (MTP 384 target plate ground steel; Bruker Daltonik) and dried. Subsequently, an aliquot (0.5 μL) of the glycan solution was spotted onto the matrix crystal and dried.

## Results

### Expression and purification of *P. minimum* IPase

**Figure 2:**
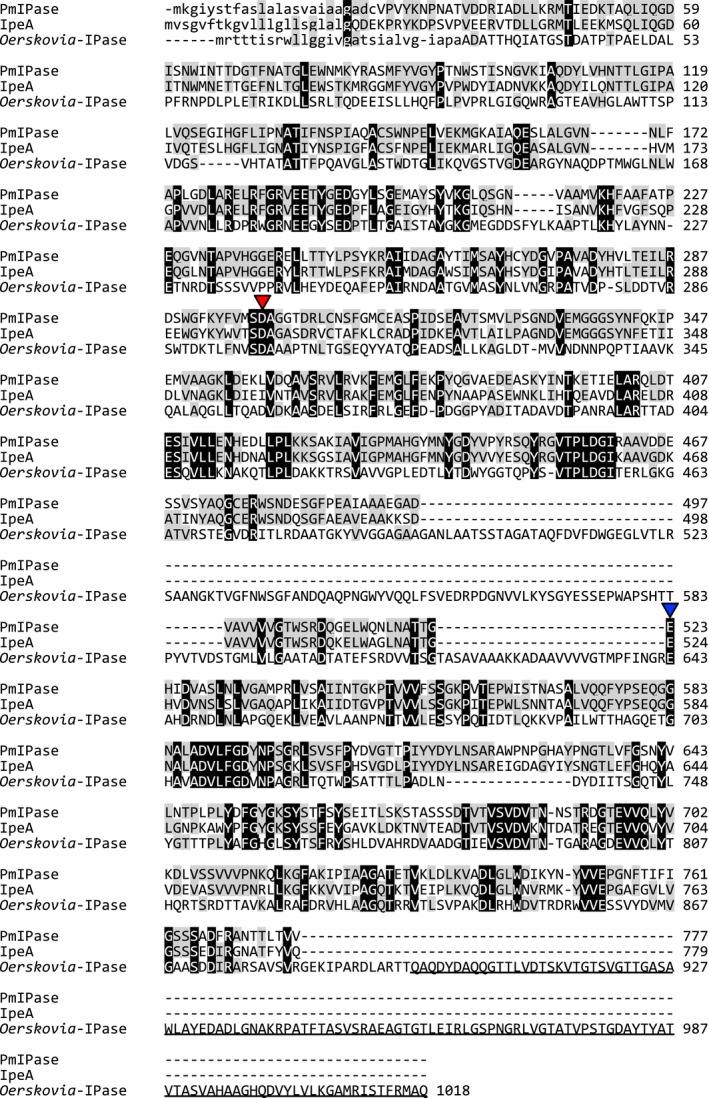
Amino acid sequence alignment of IPases. The amino acid sequences of PmIPase, *Aspergillus oryzae* IpeA, and *Oerskovia* sp. IPase were compared in ClustalW (DNA Data Bank of Japan, http://www.ddbj.nig.ac.jp/) using default settings. Conserved amino acid residues in all three IPases are shown in black, and those conserved in two IPases are shown in gray. Catalytic nucleophile and acid/base residues are indicated by red and blue triangles, respectively. The CBM6 region of *Oerskovia* sp. IPase is underlined. The putative signal peptides are indicated with lowercase letters.

*Phaeoacremonium minimum* IPase showed homology with *A. oryzae* IPase IpeA with an identity of 65% 15 and a weak homology with *Oerskovia* sp. IPase (identity 33%; Fig. 2). Only *Oerskovia* sp. IPase had a cellulose‐binding module family 6 (CBM6), whereas the fungal IPases, PmIPase, and IpeA lack a CBM. Putative catalytic residues, Asp‐299 (nucleophile) and Glu‐523 (acid/base) residues of PmIPase, were conserved among these IPases.

**Figure 3:**
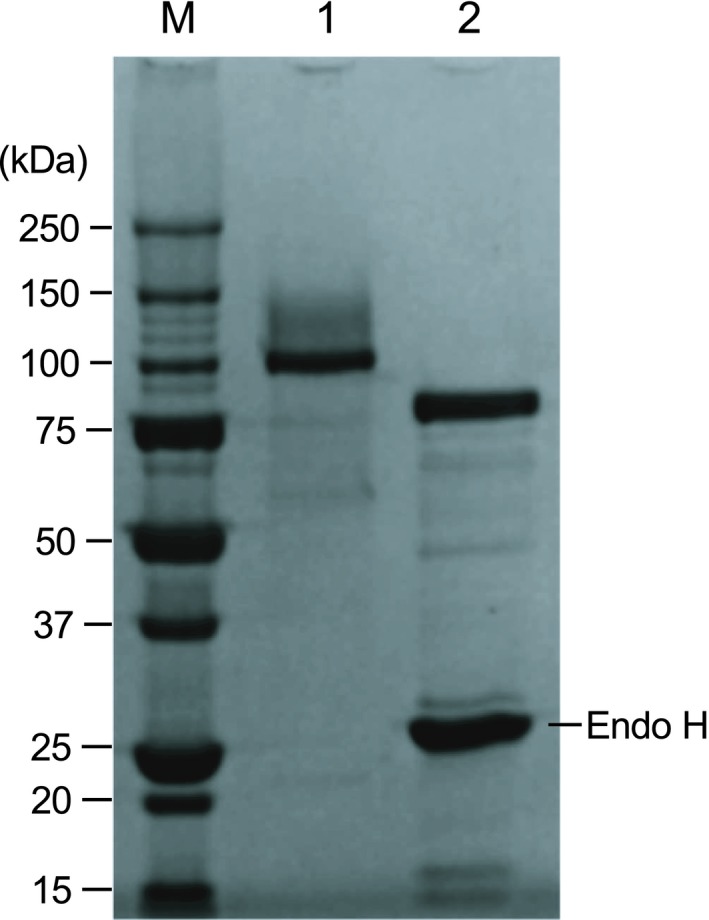
SDS/PAGE analyses of purified PmIPase. Lane M: molecular marker, lane 1: purified recombinant PmIPase, lane 2: recombinant PmIPase treated with endo H.

*Phaeoacremonium minimum* isoprimeverose‐producing enzyme expressed in *P. pastoris* and purified as described above. The gene encoding PmIPase consisted of 2334 bp and translated to PmIPase consisting of 777 amino acids. Based on an amino acid sequence analysis using signalp 4.1 (www.cbs.dtu.dk/services/SignalP), PmIPase was predicted to have an N‐terminal 24‐amino‐acid signal peptide (Fig. 2). The molecular mass of Myc‐ and His_6_‐tagged PmIPase (without the predicted N‐terminal signal peptide) was calculated to be 85 kDa. However, SDS/PAGE analyses indicated a molecular mass of approximately 101 kDa for purified recombinant PmIPase (Fig. 3). The protein band of purified PmIPase was moved to approximately 92 kDa by treatment with endo H, indicating that the PmIPase expressed in *P. pastoris* was *N*‐glycosylated. The optimal pH range and temperature of purified PmIPase toward XXXGol were pH 4.0–4.5 and 60 °C, respectively. Thermostability experiments indicated that the purified recombinant PmIPase was stable at temperatures lower than 60 °C, but was denatured at 65 °C (data not shown).

### Substrate specificity of PmIPase

The substrate specificity of purified PmIPase toward various xyloglucan oligosaccharides, such as XG, XX, and XXXG; cellobiose; and xylobiose, was examined (Table 1). PmIPase showed hydrolytic activity toward XG but not toward cellobiose, indicating that xylose residues at the nonreducing end of oligosaccharides are essential for the hydrolytic activity of PmIPase. PmIPase did not hydrolyze LG, indicating that galactosylation of the xylose residue at the nonreducing end abolishes the PmIPase activity. PmIPase showed almost the same hydrolytic activities toward 4 mm XG and XX. In the case of 2 mm XG and XX, XX was only slightly preferred over XG, but in the case of 8 mm XG and XX, XG was only slightly preferred over XX (Table 1). In a solution containing 4 mm substrate, PmIPase exhibited approximately twofold higher hydrolytic activity toward XG than toward XXXG, and galactosylation of the xylopyranosyl residue at the second glucopyranosyl residue from the nonreducing end did not show any negative effect on PmIPase activity (Table 1).

**Table 1:** Substrate specificity of recombinant PmIPase. n.d.: not detected

Substrates	Concentration (mm)	Activity (μmol·min^−1^·mg^−1^)
XG	2	56.0 ± 2.6
	4	73.6 ± 4.3
	6	78.4 ± 6.7
	8	83.1 ± 2.4
XX	2	64.8 ± 3.0
	4	74.4 ± 3.7
	6	74.8 ± 4.1
	8	72.3 ± 3.6
XXXG	2	56.5 ± 3.6
	4	38.4 ± 0.9
	6	30.6 ± 0.8
	8	25.9 ± 1.8
XLLG	2	60.8 ± 3.3
	4	44.3 ± 0.9
	6	34.8 ± 1.8
	8	27.7 ± 1.1
XXXGol	2	66.1 ± 3.6
	4	49.0 ± 1.7
	6	39.1 ± 1.8
	8	34.1 ± 1.6
XXX	5	47.0 ± 2.4
LG	5	n.d.
Cellobiose	5	n.d.
Xylobiose	5	n.d.

The hydrolytic activity of PmIPase toward XG increased with increasing substrate concentration (Table 1). By contrast, its hydrolytic activities toward XXXG and XLLG decreased with increasing substrate concentration. For example, the hydrolytic activity of PmIPase toward XXXG at a concentration of 8 mm was less than half of that at a concentration of 2 mm (Table 1).

### Kinetics of PmIPase

The kinetic parameters of PmIPase with XXXGol were compared with those of *A. oryzae* IpeA (Table 2). The *k*_cat_ of PmIPase was about one‐sixth that of *A. oryzae* IpeA, indicating that PmIPase acts on its oligosaccharide substrates relatively slowly. However, the *K*_m_ of PmIPase was one‐thirteenth that of IpeA, and the *k*_cat_/*K*_m_ of PmIPase was about 2.3‐fold higher than that of IpeA. These results indicate that PmIPase had a much higher affinity and catalytic efficiency toward XXXG than *A. oryzae* IpeA. In this analysis, the specific activity of PmIPase toward 0.2 mm XXXGol substrate was 62.0 ± 2.6 μg·min^−1^·mg^−1^. This is discussed in greater detail below.

**Table 2:** Kinetic analysis of PmIPase toward reduced XXXG substrate

	*K*_m_ (mm)	*k*_cat_ (s^−1^)	*k*_cat_/*K*_m_ (s^−1^·mm ^−1^)
PmIPase	0.0475 ± 0.0048	101 ± 3	2126
IpeA	0.629 ± 0.051	586 ± 16	931

### Transglycosylation activity of PmIPase

**Figure 4:**
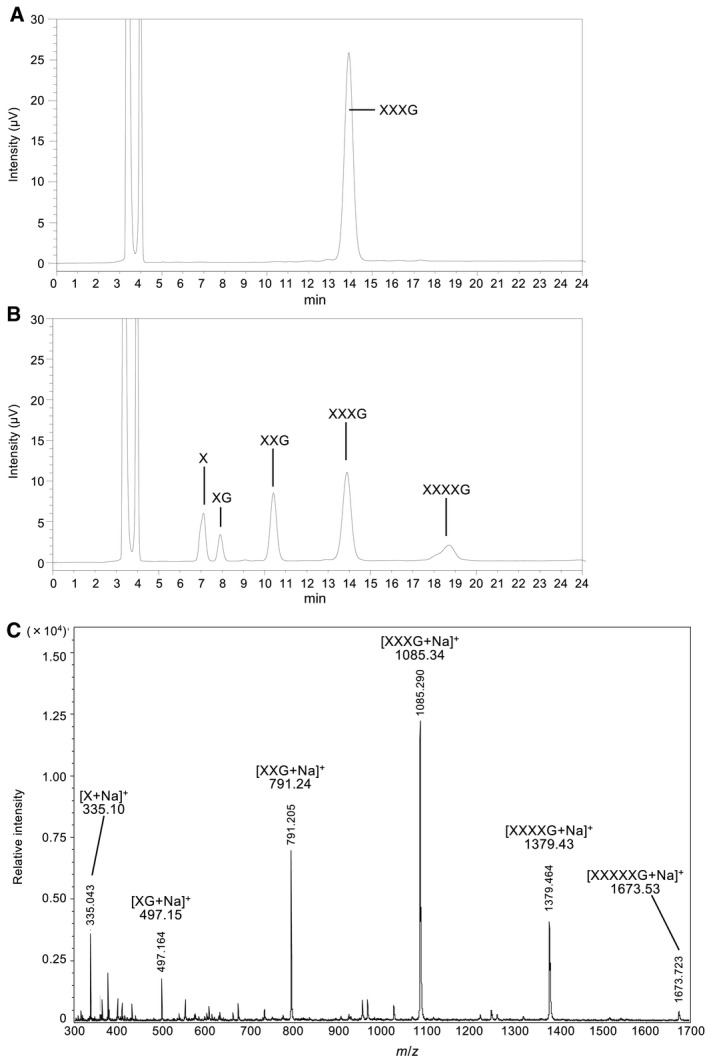
Transglycosylation activity of recombinant PmIPase. (A) XXXG substrate (8 mm) and (B) its reaction products, which were produced from XXXG by PmIPase treatment (60 °C for 5 min), were analyzed using a hydrophilic interaction chromatography. (C) The reaction products were analyzed using MALDI‐TOF mass spectrometry.

The transglycosylation activity of PmIPase was also examined. PmIPase was incubated in a high concentration (8 mm) of XXXG for 5 min. PmIPase produced not only isoprimeverose, which is a hydrolysis product, but also XXXXG and XXXXXG (Fig. 4B,C). Transglycosylation of PmIPase was also observed when PmIPase was incubated with XX (data not shown).

## Discussion

Isoprimeverose‐producing enzymes release isoprimeverose from xyloglucan oligosaccharides and are key enzymes in the production of various oligosaccharides via hydrolysis and transglycosylation. As described above, PmIPase exhibited a high transglycosylation activity in high concentrations of substrate. The observed decrease in hydrolytic activity with increasing concentrations of XXXG and XLLG may partially result from transglycosylation. The substrate specificity of PmIPase resembled that of *A*. *oryzae* IpeA. For example, both enzymes recognized the isoprimeverose unit of xyloglucan oligosaccharides at the nonreducing end, with the xylopyranosyl side chain being essential for their hydrolytic activities. Galactosylation of the xylopyranosyl side chain on the glucopyranosyl residue at the nonreducing end of xyloglucan oligosaccharide abolished all hydrolytic activity. Some differences in substrate specificity were evident between PmIPase and *A. oryzae* IpeA. *Aspergillus oryzae* IpeA preferred xyloglucan oligosaccharides containing four glucosyl main‐chain residues, such as XXXG, over substrates containing two glycosyl main‐chain residues, such as XG and XX. By contrast, PmIPase preferred substrates containing two glycosyl main‐chain residues at high substrate concentrations (4–8 mm). As described above, the specific activity of PmIPase toward 0.2 mm XXXGol substrate (0.2 mm is approximately fourfold higher than the *K*_m_ of PmIPase) was almost the same as that toward 2 mm XXXGol. In addition, the specific activities toward 4 mm XG or XX were higher than those toward 2 mm XG or XX, respectively. These results suggest that the *K*_m_ values for XG and XX were much higher than that for XXXGol and that PmIPase preferred xyloglucan oligosaccharides containing four glucosyl main‐chain residues at low substrate concentrations. The hydrolytic activity of *A. oryzae* IpeA was inhibited by galactosylation of the second xylopyranosyl residue from the nonreducing end, whereas PmIPase was unaffected. These results suggest differences in the substrate recognition abilities of positive subsites (+1, +1′, +2, etc.) 15 in PmIPase and *A. oryzae* IpeA. Future studies will focus on crystal structure analyses of IPases to elucidate the mechanisms that allow IPases to recognize xyloglucan oligosaccharide substrates at negative and positive subsites.

Based on a protein BLAST search (https://blast.ncbi.nlm.nih.gov/Blast.cgi), *P. minimum* hosts other putative enzymes related to xyloglucan degradation, including GH74 xyloglucanase (NCBI Reference Sequence: XP_007915470.1) and α‐xylosidase (NCBI Reference Sequence: XP_007915392.1). It is hypothesized that these enzymes act together with PmIPase to hydrolyze and assimilate xyloglucan into *P. minimum*.

## Author contributions

TM and KY conceived and designed the experiments. AK analyzed the oligosaccharides. TM performed and analyzed all other experiments.TM, AK, and KY wrote the paper.

## Conflict of interest

The authors declare no conflict of interest.
